# 3D Fibrin/Gelatin Hydrogel System Enhances the Therapeutic Potency of DPSC-Derived Extracellular Vesicles Compared to 2D Culture in Accelerating Diabetic Wound Healing via Angiogenesis and Immune Modulation

**DOI:** 10.3390/jfb17050244

**Published:** 2026-05-12

**Authors:** Xin Qiao, Kai Liu, Jie Tang, Shijian Deng, Deqin Yang

**Affiliations:** 1 Department of Endodontics, Shanghai Stomatological Hospital & School of Stomatology, Fudan University, Shanghai 201102, China; 2 Shanghai Key Laboratory of Craniomaxillofacial Development and Diseases, Fudan University, Shanghai 201102, China; 3 Chongqing Key Laboratory of Oral Diseases and Biomedical Sciences, Stomatological Hospital of Chongqing Medical University, Chongqing 401147, China

**Keywords:** dental pulp stem cells, extracellular vesicles, 3D fibrin/gelatin system, diabetic wound healing, angiogenesis

## Abstract

**Background**: Impaired angiogenesis and persistent inflammation are hallmarks of chronic diabetic wounds. Extracellular vesicles derived from dental pulp stem cells (DPSC-EVs) represent a promising cell-free therapy for tissue repair; however, their clinical translation is hindered by suboptimal yields and attenuated bioactivity associated with conventional two-dimensional (2D) culture. This study investigated whether a biomimetic three-dimensional (3D) fibrin/gelatin hydrogel system could optimize the therapeutic potency of DPSC-EVs for diabetic wound healing. **Methods**: DPSCs were encapsulated within 3D fibrin/gelatin scaffolds, followed by comprehensive characterization of cell viability and morphology. 3D-EVs and 2D-EVs were isolated via ultracentrifugation and validated by transmission electron microscopy and nanoparticle tracking analysis. The pro-angiogenic capacity of 3D-EVs was evaluated using human umbilical vein endothelial cells (HUVECs) under high-glucose (HG) stress. Additionally, the immunomodulatory effects were assessed by monitoring macrophage polarization in lipopolysaccharide-stimulated RAW 264.7 cells. The therapeutic efficacy was further validated in vivo using a streptozotocin (STZ)-induced diabetic mouse model with full-thickness cutaneous wounds. **Results**: The 3D fibrin/gelatin hydrogel provided a supportive microenvironment that significantly augmented the secretory productivity of DPSCs. Compared to 2D-EVs, 3D-EVs exhibited superior functional resilience in restoring HUVEC migration and tube formation under HG-induced oxidative stress. Furthermore, 3D-EVs effectively orchestrated the macrophage transition from a pro-inflammatory M1 phenotype toward an anti-inflammatory M2 phenotype, thereby modulating the immune microenvironment. In vivo, topical administration of 3D-EVs markedly accelerated wound closure, promoted re-epithelialization, and enhanced microvascular density and collagen maturation in diabetic mice. **Conclusions**: Our findings demonstrate that the 3D fibrin/gelatin culture system effectively primes the therapeutic profile of DPSC-EVs. These engineered vesicles accelerate diabetic wound healing by synergistically promoting angiogenesis and resolving chronic inflammation, offering a robust and potent cell-free strategy for the management of chronic diabetic ulcers.

## 1. Introduction

Diabetic wounds are notoriously difficult to treat and frequently recur, resulting in a prolonged healing period that places a considerable burden on healthcare systems worldwide. These persistent problems not only impair patients’ quality of life but also pose significant clinical challenges [1]. Normally, wound healing follows a precise order: inflammation, proliferation, and remodeling. However, this balance is completely disrupted in the diabetic context [2]. The core of the problem lies in the consistently hyperglycemic microenvironment, which causes chronic inflammation and immune system dysregulation [3]. While clinical studies have shown that better wound healing is achieved when HbA1c is maintained below 7%, even patients with good glycemic control can experience impaired healing due to the cumulative effects of advanced glycation end products (AGEs), microvascular dysfunction, and persistent low-grade inflammation [4]. More specifically, hyperglycemia prevents macrophages from switching from the pro-inflammatory M1 phenotype to the pro-healing M2 phenotype. As a result, an excessive amount of inflammatory cytokines is released, which continues to damage tissues [5,6]. This chronic inflammation is also closely linked to impaired blood vessel growth [7]. When neovascularization is defective, meaning endothelial cells cannot migrate or properly form vessels, the wound bed does not receive sufficient oxygen and nutrients. This ultimately prevents the healing process from progressing to the proliferative phase [8,9]. Currently, even with conventional treatments such as surgery or special dressings, results remain insufficient, often with low healing rates and high recurrence rates [10]. Therefore, it seems urgent to find new regenerative approaches to correct this hostile immune environment and promote blood vessel regrowth in order to accelerate the repair of diabetic wounds. Clinically, diabetic ulcers, as a severe form of diabetic wound, are classified as neuropathic (~30%) or neuroischemic (~70%), differing mainly in the degree of ischemia. Our model recapitulates hyperglycemia-induced microvascular dysfunction relevant to both types [11].

To overcome the limitations of traditional treatments, cell-free strategies, especially those utilizing extracellular vesicles (EVs), have received increasing attention in recent years [12]. These naturally occurring nanoscale vesicles play a key role in intercellular communication by transporting proteins, lipids, and nucleic acids to target cells [13,14]. Compared with direct stem cell transplantation, extracellular vesicle therapy offers significant clinical advantages, including a reduced risk of immune rejection, easier long-term preservation, and the elimination of risks such as tumor formation or vascular occlusion [15,16]. Recent studies have found that extracellular vesicles derived from mesenchymal stem cells accelerate the healing of diabetic wounds by inhibiting NET-induced ferroptosis in endothelial cells [17]. In addition, injectable hydrogels using extracellular vesicles derived from foreskin mesenchymal stem cells have achieved significant success in the treatment of skin wounds in chronic diabetes by continuously releasing bioactive substances [18]. Among various cell sources, dental pulp stem cells (DPSCs) have become a promising candidate cell in the field of tissue engineering, mainly due to their ease of acquisition from extracted teeth, rapid proliferation, and strong multi-lineage differentiation potential [19]. Studies have confirmed that DPSC-EVs have powerful pro-angiogenic and immunomodulatory functions, and are effective in promoting skin repair and regulating immune responses [20,21,22].

Traditional two-dimensional culture platforms cannot successfully simulate the complex three-dimensional spatial structure and rigidity of the biomimetic extracellular matrix (ECM) in vivo [23]. Cells in two-dimensional environments often undergo significant phenotypic drift and metabolic changes, resulting in insufficient molecular diversity of their secretome to support complex tissue repair [24]. The yield of extracellular vesicles collected from two-dimensional monolayer cultures is usually unsatisfactory, and their functional resilience is impaired in the adverse environment of diabetic wounds [25]. To address the inherent problems of traditional two-dimensional culture, developing three-dimensional biomimetic platforms to provide the necessary spatial and mechanical signals for regulating the cell secretome has become a key strategy. Studies have shown that three-dimensional environments can encourage stem cells to integrate more anti-apoptotic and pro-angiogenic factors into their extracellular vesicles, thereby improving their stability and therapeutic efficacy [26,27,28].

Fibrin and gelatin composite hydrogels provide a particularly favorable synergistic environment for dental pulp stem cells. Fibrin is a natural clotting protein with numerous binding sites that promote cell adhesion and can form a temporary matrix similar to the initial stage of wound healing [29]. However, pure fibrin scaffolds often degrade too quickly and lack mechanical rigidity [30]. As a collagen derivative, gelatin effectively addresses these issues through enhanced structural stability of the scaffold and the preservation of important cellular sequences, such as matrix metalloproteinase (MMP) sensitive sites [31]. This hybrid system allows researchers to tune viscoelasticity and porous structures to optimize the cellular microenvironment. The specific stiffness of these scaffolds can trigger mechanotransduction pathways via the cytoskeleton, a process associated with increased integration of pro-angiogenic and anti-inflammatory microRNAs (miRNAs) in extracellular vesicles [32]. Therefore, this study hypothesized that the 3D fibrin/gelatin environment acts as a functional bioreactor. It helps dental pulp stem cells produce extracellular vesicles with enhanced therapeutic resilience and an increased capacity to combat the hostile environment of diabetic wounds.

In the present study, we first optimized the formulation of the fibrin and gelatin hydrogel to establish a stable and biomimetic three-dimensional culture system for dental pulp stem cells. We then isolated extracellular vesicles from this engineered microenvironment and systematically compared their physical characteristics and therapeutic potential against those derived from conventional two-dimensional cultures. The biological effects of these DPSC-EVs were evaluated through a series of in vitro assays involving high-glucose-induced endothelial cell dysfunction and macrophage polarization to simulate the hostile diabetic wound environment. Furthermore, the regenerative capacity of the 3D-cultured DPSC-EVs was validated using a streptozotocin-induced diabetic mouse model to observe full-thickness wound closure, re-epithelialization, and neovascularization. Our findings demonstrate that the three-dimensional culture approach significantly enhances the angiogenic and immunomodulatory properties of DPSC-derived vesicles, offering a promising cell-free therapeutic strategy for the management of chronic diabetic wounds.

## 2. Materials and Methods

### 2.1. Cell Culture and Characterization

#### 2.1.1. Isolation and Culture of Human DPSCs

DPSCs were isolated from extracted third molars of healthy donors (*n* = 8 teeth from 5 donors; age range 18–25 years) at the Surgical Outpatient Department of Stomatological Hospital, Chongqing Medical University, with informed consent obtained. Pulp tissues from multiple teeth were pooled for cell isolation. The study was approved by the Ethics Committee (CQHS-REC-2023 (LSNo.187)). Briefly, the dental pulp tissue was extracted and digested with 3 mg/mL Type I collagenase (Sigma-Aldrich, St. Louis, MO, USA) and 4 mg/mL dispase (Roche, Basel, Switzerland) for 30 min at 37 °C. The resulting cell suspension was cultured in α-MEM (Gibco, Grand Island, NY, USA) supplemented with 10% fetal bovine serum (FBS; Gibco, Grand Island, NY, USA) and 1% penicillin/streptomycin (Hyclone, Logan, UT, USA). Cells from passages 3 to 5 were used for all experiments to ensure phenotypic stability.

#### 2.1.2. Culture of HUVECs and RAW 264.7 Cells

Human umbilical vein endothelial cells (HUVECs) and the murine macrophage cell line RAW 264.7 were purchased from the American Type Culture Collection (ATCC, Manassas, VA, USA). HUVECs were maintained in Endothelial Cell Medium (ECM; ScienCell, Carlsbad, CA, USA), while RAW 264.7 cells were cultured in high-glucose DMEM (Gibco, Grand Island, NY, USA) supplemented with 10% FBS. All cells were incubated in a humidified atmosphere with 5% CO_2_ at 37 °C.

### 2.2. Preparation and Characterization of Fibrin/Gelatin (FG) Hydrogels

#### 2.2.1. Synthesis of the 3D FG Hydrogel Scaffold

The 3D biomimetic hydrogel was prepared by cross-linking fibrinogen and gelatin in an optimized ratio (GF 1:1) to support DPSC growth and EV secretion. Fibrinogen (Sigma-Aldrich, St. Louis, MO, USA) and gelatin (Sigma-Aldrich, St. Louis, MO, USA) were dissolved in phosphate-buffered saline (PBS; HyClone, Logan, UT, USA) to reach the target concentrations (5 mg/mL for fibrinogen and 4% *w*/*v* for gelatin). The precursor solution was then mixed with 0.5 U/mL thrombin (Sigma-Aldrich, St. Louis, MO, USA) and 20 mM CaCl_2_ (Sigma-Aldrich, St. Louis, MO, USA) to initiate rapid gelation, which typically occurred within 6 min at 37 °C. The hydrogel precursor solution (200 μL per scaffold) was cast into 48-well plates to form cylindrical scaffolds with a diameter of approximately 11 mm and a height of approximately 2 mm. The final volume of each hydrogel scaffold was approximately 200 μL. For 3D cell encapsulation, DPSCs were suspended in the precursor solution at a density of 1 × 10^6^ cells/mL prior to cross-linking. The cell-laden hydrogels were then cultured in FBS-free medium to avoid exogenous vesicle contamination.

#### 2.2.2. Rheological Characterization

The rheological properties of hydrogels with different Gelatin-to-Fibrin volume ratios (GF 1:3, 1:2, 1:1, and 2:1) were characterized using a rotational rheometer (TA Instruments, New Castle, DE, USA) with a 20 mm parallel plate geometry. Time-sweep oscillatory tests were performed at 37 °C (1 Hz, 1% strain) to monitor the storage modulus (G′) and loss modulus (G″), and gelation time was determined as the point of G′/G″ crossover or when G′ reached a plateau. Frequency-sweep tests (0.1–100 rad/s, 1% strain, 37 °C) were then conducted on fully cross-linked hydrogels to compare their mechanical rigidity. All measurements were performed in triplicate.

#### 2.2.3. Morphological and Porosity Analysis

The internal microstructure of the optimized GF 1:1 hydrogel was examined using Scanning Electron Microscopy (SEM; Hitachi, Tokyo, Japan). The hydrogel samples were fixed with 2.5% glutaraldehyde (Sigma-Aldrich, St. Louis, MO, USA) at 4 °C for 24 h, rinsed with PBS, and dehydrated through a graded ethanol series. Subsequently, the samples were lyophilized in a freeze-dryer (Labconco, Kansas City, MO, USA) and sputter-coated with gold (Quorum Technologies, Laughton, UK) for observation. To quantify the pore size, twenty different fields of view were randomly selected from the SEM images, and the diameters were measured using ImageJ software v. 1.54 (National Institutes of Health, Bethesda, MD, USA) to evaluate their suitability for nutrient exchange and EV diffusion.

#### 2.2.4. In Vitro Degradation Assay

The biodegradation rate of the GF 1:1 hydrogel was evaluated by measuring the residual weight of the samples over time. PBS was used as a standardized medium to assess baseline hydrolytic degradation, providing reproducible conditions for comparing scaffold stability across formulations. Briefly, the cross-linked hydrogels (*n* = 3) were weighed (*W*_0_) and then immersed in 2 mL of PBS. The samples were incubated at 37 °C with gentle agitation. At predetermined time points (1, 3, 5, 7, 10, and 14 days), the hydrogels were removed, gently blotted with filter paper to remove surface water, and weighed (*W_t_*). The degradation percentage was calculated using the following formula:

$$Degradation\;Rate\;(\%)=\frac{\left(W_{0}-W_{t}\right)}{W_{0}}\times 100\%$$

### 2.3. Morphology and Viability of DPSCs in 3D Culture

#### 2.3.1. SEM Observation of Cell Morphology

To evaluate the initial attachment and morphology of DPSCs within the 3D microenvironment, DPSCs were encapsulated in the GF 1:1 hydrogel at a density of 1 × 10^6^ cells/mL and cultured for 24 h. Subsequently, the cell-laden hydrogels were collected and processed for Scanning Electron Microscopy (SEM; Hitachi, Tokyo, Japan) observation. The samples were fixed, dehydrated, and lyophilized according to the procedures described in Section 2.2.3.

#### 2.3.2. Live/Dead Staining and Cytocompatibility Analysis

The cytocompatibility of the hydrogel was evaluated using a Calcein-AM/PI Live/Dead Cell Viability Assay Kit (Beyotime Biotechnology, Shanghai, China). Briefly, after 3 and 7 days of 3D culture, the cell-laden hydrogels were washed with PBS and incubated with the staining solution for 30 min at 37 °C in the dark. Live cells (green) and dead cells (red) were visualized using a confocal laser scanning microscope (CLSM; Leica, Wetzlar, Germany). To quantify cell viability, the number of live and dead cells from five random fields per sample was counted using ImageJ software (NIH, Bethesda, MD, USA), and the results were expressed as the ratio of dead cells to live cells (Dead/Live ratio) to evaluate the long-term biocompatibility of the scaffold.

### 2.4. Isolation and Characterization of DPSC-EVs

#### 2.4.1. Collection and Isolation of 2D- and 3D-Cultured DPSC-EVs

To collect EVs, DPSCs were cultured in either conventional 2D culture plates or 3D GF 1:1 hydrogels. When the 2D culture reached 80% confluence, the growth medium was replaced with serum-free α-MEM. Similarly, cell-laden 3D hydrogels were maintained in serum-free medium. The conditioned media from both groups were collected every 24 h for 7 consecutive days and stored at −80 °C. The EVs were isolated using a series of differential centrifugation steps at 4 °C. Briefly, the collected media were centrifuged at 300× *g* for 10 min, 3000× *g* for 10 min, and 10,000× *g* for 30 min to remove cells, dead cells, and cellular debris, respectively. The supernatant was then ultracentrifuged at 100,000× *g* for 70 min (Beckman Coulter, Brea, CA, USA) to pellet the EVs. The resulting pellets were resuspended in phosphate-buffered saline (PBS; HyClone, Logan, UT, USA) and subjected to a second round of ultracentrifugation at 100,000× *g* for 70 min for purification. The final EV pellets were resuspended in PBS and stored at −80 °C for further use. According to MISEV2023 guidelines, our isolated EVs exhibit characteristics of small extracellular vesicles (50–150 nm, CD63^+^/TSG101^+^), consistent with an exosome-enriched population. However, due to the use of ultracentrifugation, we refer to them as ‘EVs’ throughout the manuscript to acknowledge potential heterogeneity.

#### 2.4.2. Morphological Characterization via TEM

The morphology of 2D-EVs and 3D-EVs was observed using Transmission Electron Microscopy (TEM; Hitachi, Tokyo, Japan). Briefly, EV suspensions were dropped onto copper grids and allowed to adsorb for 5 min. The grids were then negatively stained with 2% uranyl acetate for 2 min, followed by air-drying at room temperature before imaging.

#### 2.4.3. Particle Size Analysis via NTA

The size distribution and particle concentration of the EVs were measured using Nanoparticle Tracking Analysis (NTA) with a ZetaView system (Particle Metrix, Meerbusch, Germany). The EV samples were diluted with PBS to an appropriate concentration, and the movement of particles was recorded and analyzed to determine the diameter and concentration.

#### 2.4.4. Western Blot Analysis of EV Surface Markers

The expression of specific EV protein markers was detected by Western blot. Total protein was extracted from the EV samples using RIPA lysis buffer. Equal amounts of protein were separated by SDS-PAGE and transferred onto PVDF membranes (Millipore, Billerica, MA, USA). After blocking with 5% non-fat milk, the membranes were incubated with primary antibodies against CD63 and TSG101; all antibodies from Abcam, Cambridge, UK) overnight at 4 °C. The membranes were then incubated with HRP-conjugated secondary antibodies and visualized using an ECL detection system (Bio-Rad, Hercules, CA, USA).

### 2.5. In Vitro Angiogenesis Assays

#### 2.5.1. EV Uptake Assay

To verify the internalization of 3D-EVs by HUVECs, the EVs were labeled with the red fluorescent membrane dye PKH26 (Sigma-Aldrich, St. Louis, MO, USA) according to the manufacturer’s instructions. Briefly, the EV suspension was incubated with PKH26 for 5 min, and the labeling reaction was terminated by adding 1% bovine serum albumin (BSA). To remove excess dye, the labeled 3D-EVs were purified by ultracentrifugation at 100,000× *g* for 70 min at 4 °C. HUVECs were then incubated with the labeled 3D-EVs for 12 h at 37 °C. Subsequently, the cells were stained with Calcein-AM (Beyotime Biotechnology, Shanghai, China) to visualize the cytoplasm (green) and fixed with 4% paraformaldehyde. The nuclei were counterstained with DAPI (Beyotime Biotechnology, Shanghai, China). The intracellular localization and uptake of 3D-EVs were observed and captured using a confocal laser scanning microscope (CLSM; Leica, Wetzlar, Germany).

#### 2.5.2. Cell Proliferation Assay

The effect of 3D-EVs on HUVEC proliferation and the determination of the optimal working concentration were evaluated using a CCK-8 assay kit (Beyotime Biotechnology, Shanghai, China). HUVECs were seeded in 96-well plates at a density of 5 × 10^3^ cells per well. After 24 h of starvation in serum-free medium, the cells were treated with 3D-EVs at varying particle concentrations: 0 (control), 1 × 10^7^, 5 × 10^7^, 1 × 10^8^, and 5 × 10^8^ particles/mL. At 12 h and 24 h of incubation, 10 μL of CCK-8 reagent was added to each well and incubated for an additional 2 h at 37 °C. The absorbance was measured at 450 nm using a microplate reader (Bio-Rad, Hercules, CA, USA) to identify the most effective concentration for subsequent functional assays.

#### 2.5.3. Wound Healing Assay

HUVEC migration under high-glucose conditions was evaluated using a wound healing assay. Cells were seeded in 6-well plates and cultured until full confluence. To simulate a diabetic microenvironment, the cells were pre-treated with high-glucose medium (containing 33.3 mM D-glucose; Sigma-Aldrich, St. Louis, MO, USA) for 24 h. A sterile 200 μL pipette tip was then used to create a uniform linear scratch in the cell monolayer. After washing with PBS to remove detached cells and debris, the HUVECs were randomly assigned to three groups and incubated in serum-free high-glucose medium supplemented with PBS (control group), 2D-EVs, or 3D-EVs. Images of the denuded area were captured at 0, 12, and 36 h post-scratching using an inverted microscope (Olympus, Tokyo, Japan). The migration capacity was quantified by measuring the area of the wound using ImageJ software. The migration rate was calculated according to the following formula:

$$Migration\;Rate\;(\%)=\frac{\left(A_{0}-A_{t}\right)}{A_{0}}\times 100\%$$

where *A*_0_ represents the initial scratch area at 0 h, and *A_t_* represents the remaining wound area at 12 h or 36 h.

#### 2.5.4. Transwell Migration Assay

The chemotactic migration of HUVECs under diabetic conditions was further assessed using Transwell chambers with 8 μm pore size filters (Corning, NY, USA). Prior to the assay, HUVECs were cultured in high-glucose medium (33.3 mM) for 24 h. Subsequently, 2 × 10^4^ cells suspended in 200 μL of serum-free high-glucose medium were seeded into the upper chamber. The lower chamber was filled with 600 μL of high-glucose medium supplemented with PBS, 2D-EVs, or 3D-EVs. After 12 h of incubation at 37 °C, cells that had migrated to the lower surface of the membrane were fixed with 4% paraformaldehyde and stained with 0.1% crystal violet (Beyotime Biotechnology, Shanghai, China). The non-migrated cells on the upper surface were gently removed with a cotton swab. The number of migrated cells was quantified by counting five random fields per well using an inverted microscope.

#### 2.5.5. Tube Formation Assay

To evaluate the angiogenic potential of HUVECs in a simulated diabetic microenvironment, a tube formation assay was performed on Matrigel (Corning, NY, USA). The 48-well plates were coated with 150 μL of Matrigel per well and allowed to polymerize at 37 °C for 30 min. HUVECs, pre-treated with high-glucose medium (33.3 mM), were seeded onto the Matrigel and treated with PBS, 2D-EVs, or 3D-EVs in high-glucose conditions. After 12 h of incubation, the formation of capillary-like structures was observed and photographed using an inverted microscope (Olympus, Tokyo, Japan). The total tube length and the number of nodes/junctions were quantified using the Angiogenesis Analyzer plugin in ImageJ software.

### 2.6. In Vitro Macrophage Polarization Assays

#### 2.6.1. Polarization Induction and Treatment Grouping

To simulate a diabetic inflammatory microenvironment, RAW 264.7 cells were cultured in high-glucose medium (33.3 mM) and primed with 100 ng/mL Lipopolysaccharide (LPS; Sigma-Aldrich, St. Louis, MO, USA) for 24 h to induce M1 polarization. Subsequently, the cells were randomly assigned to PBS (control), 2D-EVs, or 3D-EVs treatment groups in high-glucose conditions. After an additional 24 h of incubation, the cells were harvested for downstream gene and protein expression analysis.

#### 2.6.2. Gene Expression Analysis via RT-qPCR

Total RNA was extracted from treated RAW 264.7 cells using TRIzol reagent (Thermo Fisher Scientific, Waltham, MA, USA). After determining RNA concentration and purity, complementary DNA (cDNA) was synthesized using a reverse transcription kit (Takara, Tokyo, Japan). Quantitative PCR was performed using SYBR Green Master Mix (Roche, Basel, Switzerland) on a real-time PCR system (Bio-Rad, Hercules, CA, USA). The mRNA expression levels of the M1-related marker (*CD86*) and M2-related markers (*Arg1*, *CD163*, and *CD206*) were analyzed. The primer sequences are shown in Table 1. Target gene expressions were normalized to GAPDH using the 2^−ΔΔCt^ method.

#### 2.6.3. Flow Cytometry Analysis

To quantify the macrophage phenotypes, cells were harvested, washed with PBS, and blocked with anti-mouse CD16/32. For M1 polarization analysis, the cells were co-stained with FITC-conjugated anti-mouse F4/80 and APC-conjugated anti-mouse CD86. For M2 polarization analysis, the cells were co-stained with FITC-conjugated anti-mouse F4/80 and APC-conjugated anti-mouse CD206 (all antibodies from BioLegend, San Diego, CA, USA). After incubation for 30 min at 4 °C in the dark, the samples were analyzed using a flow cytometer (BD Biosciences, San Jose, CA, USA). The percentage of F4/80^+^ CD86^+^ (M1) and F4/80^+^ CD206^+^ (M2) populations was determined using FlowJo software v10.8.

### 2.7. In Vivo Diabetic Wound Healing Study

#### 2.7.1. Establishment of Diabetic Mouse Model

All animal procedures were approved by the Animal Ethics Committee of Shanghai Jiao Tong University Institutional Animal Care and Use Committee (IACUC) Ethics for Animal Protocols (NO. A2025266). The modeling method refers to previous reports [33]. Male C57BL/6 mice (8–10 weeks old) were used. To induce diabetes, mice were fasted for 12 h with free access to water and then received a single intraperitoneal injection of 2% STZ (Sigma-Aldrich, St. Louis, MO, USA; 150 mg/kg body weight) freshly dissolved in 0.05 M citrate buffer (pH 4.5). After STZ injection, blood glucose levels were monitored for three consecutive days to ensure initial elevation. After one week, mice with a fasting blood glucose level ≥ 16.67 mmol/L for three consecutive times were considered successful diabetic models and used for subsequent experiments. Their body weights were monitored throughout the study. The sample size for the in vivo study was determined based on a power analysis using the primary outcome measure (wound healing rate at day 5). From our preliminary experiments, the mean healing rate in the control group was 35% with a standard deviation of 12%, and we anticipated a minimal clinically relevant difference of 30% in the 3D-EVs group. Using a two-sided significance level (α) of 0.05 and a statistical power (1−β) of 0.80, a minimum of 5 mice per group was calculated using G*Power software (version 3.1). To account for potential dropouts (due to unexpected death or wound infection), 6 mice per group were enrolled. All animals that completed the study were included in the final analysis with no exclusions.

#### 2.7.2. Full-Thickness Skin Wound Model and Treatment

After successful diabetes induction, mice were randomly assigned to three experimental groups (PBS, 2D-EVs, and 3D-EVs) using a computer-generated random number sequence (www.random.org). The allocation sequence was generated by an independent researcher who was not involved in animal handling or outcome assessment. Group allocation was concealed using sequentially numbered, opaque, sealed envelopes, which were opened only immediately before wound creation and treatment administration. The animal housing, wound creation, and treatment administration were performed by one investigator (X.Q.), while outcome assessments (wound photography, histological and immunofluorescence analyses, and quantification of wound healing rates) were conducted by a second investigator (K.L.) who was blinded to the group allocation. All data analyses were performed with the investigator blinded to the treatment groups. The blinding code was broken only after the final statistical analysis was completed. Under anesthesia and standard aseptic conditions, the dorsal hair of diabetic mice was shaved, and a full-thickness excision wound with a 5 mm diameter was created on the back using a sterile biopsy punch (Miltex, York, PA, USA). The diabetic mice were randomly assigned to three groups and received local injections around the wound margin. Each wound received 100 μL of either PBS (control), 2D-EVs, or 3D-EVs (all resuspended in PBS at a concentration of 5 × 10^8^ particles/mL), administered via four equally distributed injections (25 μL per site) using a 30-gauge insulin syringe. The wounds were photographed at day 0, 5, and 10 post-surgery to monitor the healing process. The wound area was quantified using ImageJ software, and the healing rate was calculated as follows:

$$Healing\;Rate\;(\%)=\frac{\left(A_{0}-A_{t}\right)}{A_{0}}\times 100\%$$

where *A*_0_ is the original wound area at day 0, and *A_t_* is the wound area at day 5 or 10.

#### 2.7.3. Histological and Immunofluorescence Analysis

At day 10, mice were sacrificed, and the full-thickness wound skin, including surrounding tissues, was collected and fixed in 4% paraformaldehyde (Sigma-Aldrich, USA). The tissues were paraffin-embedded, sectioned at 5 μm, and stained with Hematoxylin and Eosin and Masson’s trichrome for histological evaluation. For immunofluorescence, sections were blocked with 5% goat serum and incubated with primary antibodies against CD31 (1:200; Abcam, UK) and α-SMA (1:500; Abcam, UK) for angiogenesis, or CD86 (1:100; Abcam, UK) and CD206 (1:200; Abcam, UK) for macrophage polarization. Sections were then incubated with Alexa Fluor 594- and 488-conjugated secondary antibodies (Thermo Fisher Scientific, USA), and nuclei were counterstained with DAPI (Beyotime Biotechnology, China). Images were captured using a fluorescence microscope (Olympus, Japan) or a confocal laser scanning microscope (Leica, Germany).

### 2.8. Statistical Analysis

All quantitative data in this study were collected from at least three independent biological replicates and are presented as the mean ± standard deviation (SD). Statistical analyses were performed using GraphPad Prism 9.0 software (GraphPad Software, San Diego, CA, USA). For comparisons between two groups, an unpaired Student’s *t*-test was employed. For comparisons among three or more groups, a one-way analysis of variance (ANOVA) was used, followed by Tukey’s post hoc test for multiple comparisons. A *p*-value < 0.05 was considered statistically significant (* *p* < 0.05, ** *p* < 0.01, *** *p* < 0.001, and **** *p* < 0.0001).

## 3. Results

### 3.1. Optimization and Characterization of the FG Hybrid Hydrogel

**Figure 1:**
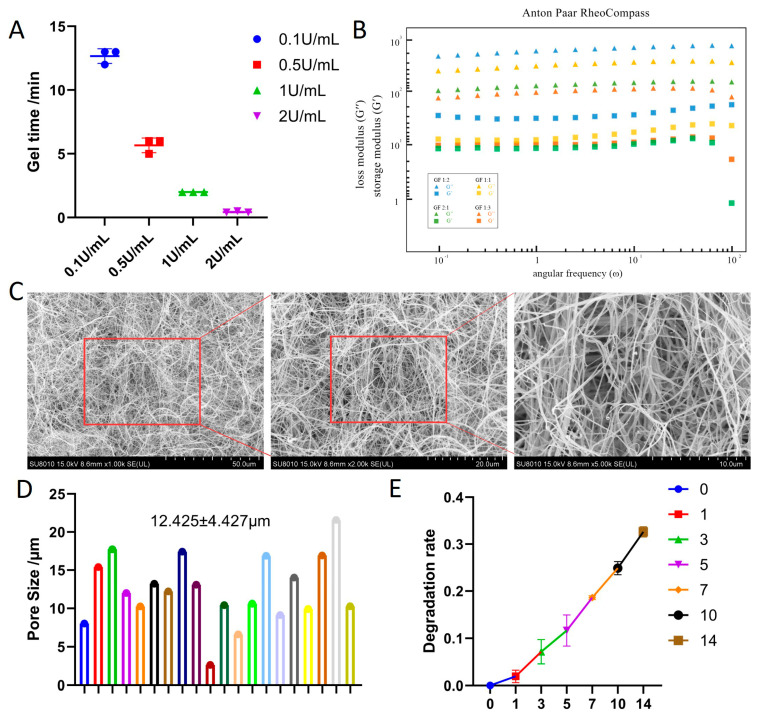
Preparation and characterization of the Fibrin/Gelatin (FG) hybrid hydrogel. (**A**) Optimization of gelation time as a function of thrombin concentration (0.1–2.0 U/mL). (**B**) Rheological characterization (Storage modulus G′ and Loss modulus G″) of FG hydrogels with varying volume ratios (GF 1:3 to GF 2:1). (**C**) Representative SEM images showing the interconnected microporous architecture of the GF 1:1 hydrogel. (**D**) Statistical analysis of the pore size distribution based on 20 random fields of view, showing an average diameter of 12.425 ± 4.427 μm. (**E**) In vitro degradation curve of the GF 1:1 hydrogel in PBS at 37 °C over 14 days, demonstrating sustained structural integrity.

To establish a biomimetic three-dimensional microenvironment for dental pulp stem cell culture and subsequent extracellular vesicle enrichment, we first optimized the thrombin concentration and component ratio of the fibrin/gelatin (FG) hydrogel. First, gelation kinetics were evaluated by measuring coagulation time at a series of thrombin concentrations (Figure 1A), showing a typical dose-dependent decrease in coagulation time. A thrombin concentration of 0.5 U/mL provided the optimal working window (approximately 6 min) for subsequent experiments. Oscillatory rheology was used to investigate the effect of different gelatin to fibrin (GF) volume ratios on the viscoelasticity of the hydrogel (Figure 1B). The storage modulus (G′) of all tested groups was consistently higher than the loss modulus (G″), indicating the formation of a stable, predominantly elastic network. The GF 1:1 matrix provided a balance between mechanical stability and biomimetic compliance, ensuring sufficient structural integrity to withstand repeated culture medium replacements while maintaining an appropriate porous structure to promote cell spreading and efficient diffusion of extracellular vesicles.

The microstructure of the optimized GF 1:1 hydrogel was observed using scanning electron microscopy (Figure 1C). The hydrogel exhibited a highly interconnected three-dimensional microporous fiber network; quantitative analysis of the pore size distribution (Figure 1D) showed an average pore size of 12.425 ± 4.427 μm. This dense yet permeable micron-sized pore structure, based on fibrin, mimics the natural extracellular matrix (ECM), providing abundant bioadhesion sites for dental pulp stem cells. Furthermore, in vitro degradation curves (Figure 1E) showed that the scaffold retained over 80% of its initial mass after 7 days and approximately 70% of its integrity after 14 days. These results confirm that the fibrin/gelatin hydrogel possesses sufficient biostability to support the continuous growth of dental pulp stem cells in a stable three-dimensional structure and long-term extracellular vesicle collection.

### 3.2. Biocompatibility and Morphology of DPSCs in 3D FG Hydrogel

**Figure 2:**
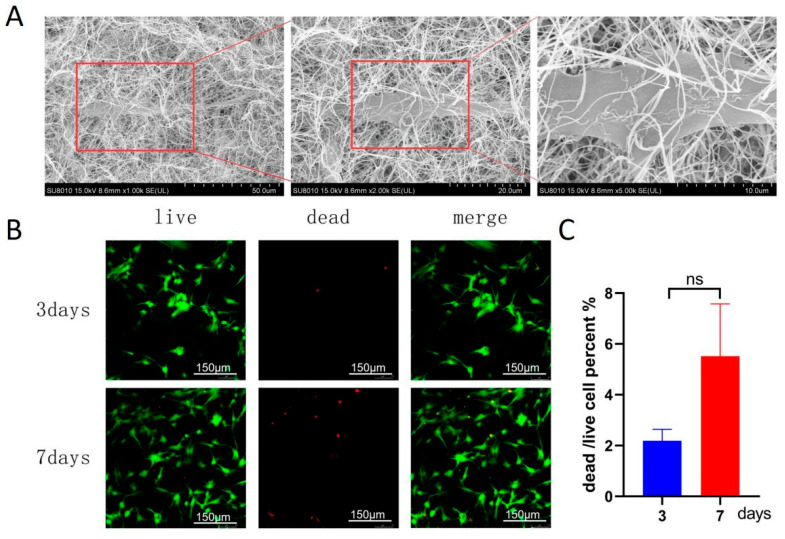
Morphology and viability of DPSCs cultured in the 3D FG hydrogel. (**A**) Representative SEM images of DPSCs encapsulated within the FG hydrogel, highlighting the spindle-shaped morphology and tight attachment to the fibrous network. (**B**) Live/Dead staining of DPSCs after 3 and 7 days of 3D culture. Green: live cells (Calcein-AM); Red: dead cells (PI). Scale bars = 150 μm. (**C**) Quantitative analysis of the dead/live cell ratio at day 3 and day 7. Data are presented as mean ± SD (*n* = 3). No statistical difference was observed between the two time points (*p* > 0.05), demonstrating the high and stable biocompatibility of the 3D scaffold. ns shows no significant.

To evaluate the suitability of the optimized fibrin/gelatin hydrogel for the culture of three-dimensional dental pulp stem cells (DPSCs), we characterized the morphology and viability of cells within the scaffold. Scanning electron microscopy revealed that DPSCs successfully integrated into the three-dimensional fiber network, exhibiting an elongated spindle-shaped morphology and extending pseudopodia to anchor on the fiber surface, indicating that the hydrogel provided sufficient bioadhesion ligands and physical support (Figure 2A). Live/dead staining results showed that the vast majority of cells remained viable on days 3 and 7, with only a very small number of dead cells observed (Figure 2B). Quantitative analysis showed a consistently low dead/live ratio, approximately 2.1% on day 3 and approximately 4.8% on day 7, with no significant difference between the two time points (*p* > 0.05) (Figure 2C). These results confirm that the fibrin/gelatin hydrogel maintained a highly biocompatible microenvironment, supporting the continuous survival and healthy proliferation of DPSCs in the three-dimensional structure, laying a solid foundation for subsequent collection and functional analysis of DPSC-EVs cultured in three dimensions. We did not assess cell viability at daily intervals; however, the stable viability from day 3 to day 7 and the sustained EV yield over 7 days support the biocompatibility of the 3D hydrogel.

### 3.3. Isolation and Characterization of 2D-EVs and 3D-EVs

**Figure 3:**
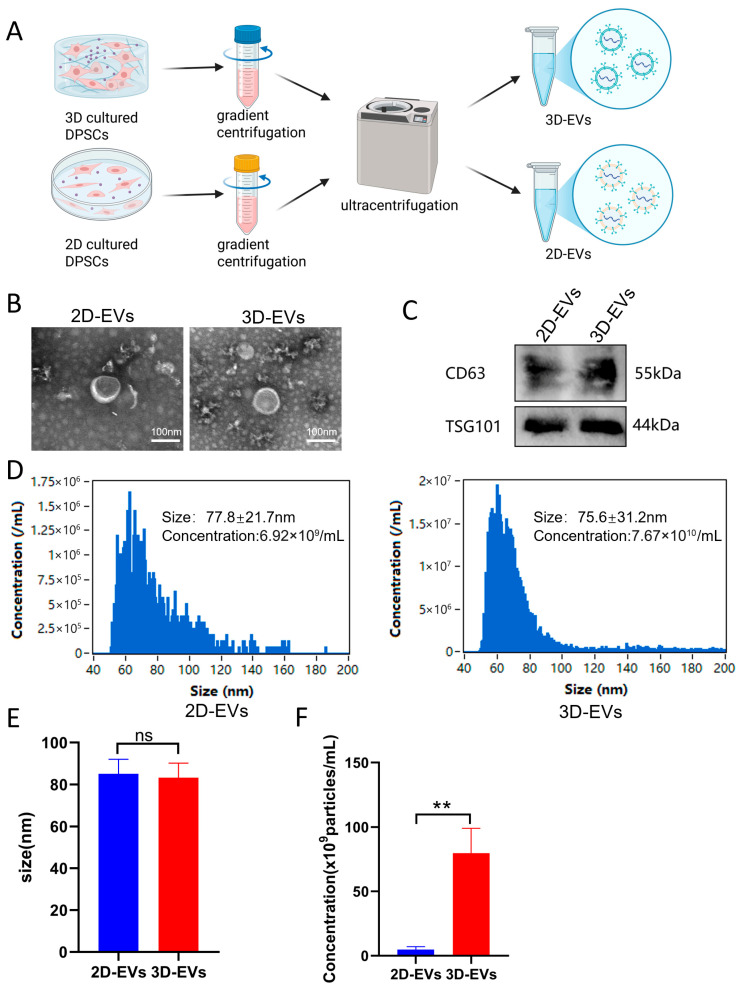
Characterization and quantitative comparison of 2D-EVs and 3D-EVs. (**A**) Schematic illustration of the workflow for isolating EVs from conventional 2D culture and 3D FG hydrogel culture. (**B**) Representative TEM images of 2D-EVs and 3D-EVs showing the characteristic cup-shaped morphology. Scale bars = 100 nm. (**C**) Western blot analysis of exosomal markers CD63 and TSG101. (**D**) Size distribution and particle concentration measured by Nanoflow cytometry (nFCM). (**E**) Statistical comparison of the mean particle size between 2D-EVs and 3D-EVs (*n* = 3, ns shows no significant). (**F**) Comparison of EV concentrations normalized to the same initial cell number, showing a significantly higher yield in the 3D group (*n* = 3,** *p* < 0.01).

To compare the secretory profiles of DPSCs under different culture conditions, we isolated extracellular vesicles from conventional 2D cultures (2D-EVs) and FG hydrogel-based 3D cultures (3D-EVs) (Figure 3A). Transmission electron microscopy (TEM) revealed that both 2D-EVs and 3D-EVs exhibited typical cup-shaped morphology with intact membrane structures (Figure 3B). Western blot analysis confirmed the presence of exosomal positive markers CD63 and TSG101 in both groups (Figure 3C). The particle size and concentration were further quantified using Nanoflow cytometry (nFCM) (Figure 3D). The size distribution curves showed that both types of EVs were primarily distributed within the range of 50–150 nm. Statistical analysis indicated no significant difference in the mean particle size between 2D-EVs and 3D-EVs (*p* > 0.05, Figure 3E), suggesting that the 3D microenvironment does not alter the fundamental physical dimensions of the secreted vesicles. Crucially, the yield of EVs was significantly affected by the culture configuration. Quantitative data showed that the concentration of 3D-EVs was substantially higher than that of 2D-EVs (*p* < 0.01, Figure 3F).

### 3.4. 3D-EVs Internalize into HUVECs and Rescue High Glucose-Impaired Angiogenic Functions In Vitro

**Figure 4:**
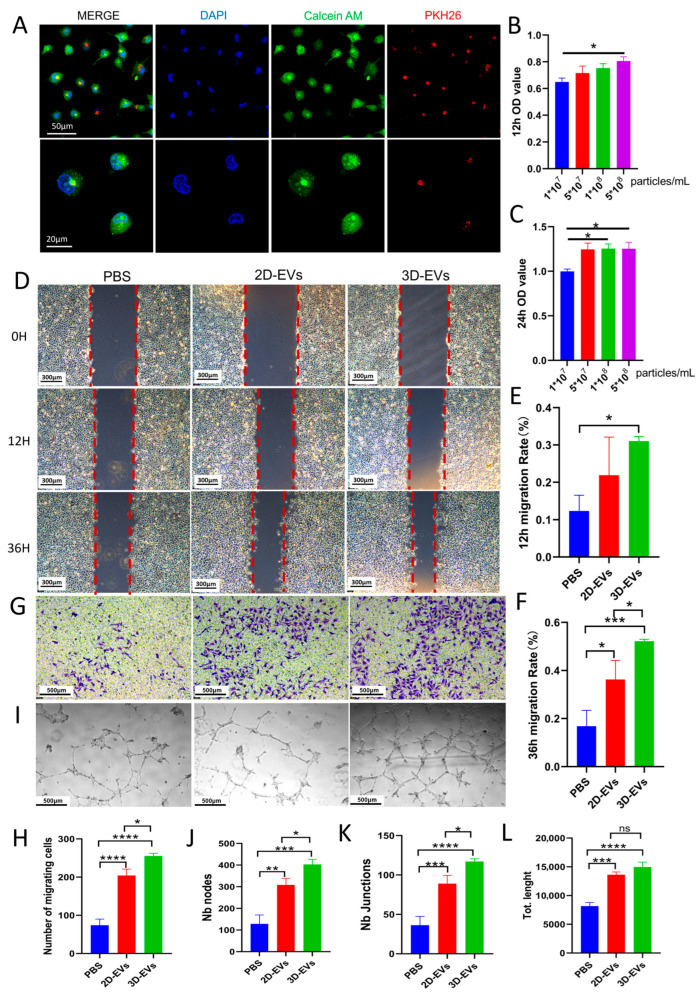
3D-EVs promote the proliferation, migration, and tube formation of HUVECs in a high-glucose (HG) environment. (**A**) Representative fluorescence images showing the cellular uptake of PKH26-labeled 3D-EVs (red) by HUVECs. Nuclei were counterstained with DAPI (blue). (**B**,**C**) Cell viability of HG-impaired HUVECs treated with varying concentrations of 3D-EVs for 12 h (**B**) and 24 h (**C**), identifying 5 × 10^8^ particles/mL as the optimal working concentration. (**D**) Representative images of the scratch wound healing assay for PBS, 2D-EVs, and 3D-EVs groups at 0 h, 12 h, and 36 h. (**E**,**F**) Quantitative analysis of the migration rate at 12 h (**E**) and 36 h (**F**). (**G**) Representative images of HUVECs migrating through the Transwell chamber membrane across different treatment groups. (**H**) Quantitative analysis of migrated cells in the Transwell assay. (**I**) Representative images of capillary-like tube formation by HUVECs on Matrigel. (**J**–**L**) Quantitative analysis of the angiogenic network, including the number of nodes (**J**), the number of junctions (**K**), and the total tube length (**L**). Data are presented as mean ± SD (*n* = 3, * *p* < 0.05, ** *p* < 0.01, *** *p* < 0.001, **** *p* < 0.0001, ns shows no significant).

To exert their biological functions, extracellular vesicles must first be internalized by target cells. We incubated PKH26-labeled 3D-EVs (red fluorescence) with HUVECs for 6 h. Fluorescent imaging confirmed the successful cellular uptake of 3D-EVs, which were predominantly localized in the perinuclear region of the HUVECs (Figure 4A). Hyperglycemia is known to severely compromise endothelial cell function, delaying the wound healing process. To evaluate the cytoprotective and proliferative effects of 3D-EVs under diabetic-like conditions, we performed a CCK-8 assay on HUVECs cultured in a high-glucose (HG) environment. Treatment with various concentrations of 3D-EVs for 12 h (Figure 4B) and 24 h (Figure 4C) demonstrated a dose-dependent promotion of cell viability. Based on these proliferation kinetics, a concentration of 5 × 10^8^ particles/mL exhibited the most robust stimulatory effect and was thus selected as the optimum working concentration for all subsequent in vitro functional assays.

Endothelial cell migration is a critical early event in angiogenesis and wound healing. We performed scratch wound healing and Transwell assays to compare the pro-migratory effects of 2D-EVs and 3D-EVs under HG conditions. As expected, the PBS-treated HG control group exhibited severely impaired migratory capacity. While treatment with conventional 2D-EVs partially attenuated this impairment, the administration of 3D-EVs demonstrated a markedly superior rescue effect (Figure 4D,G). Quantitative analysis of the scratch assay at 12 h and 36 h (Figure 4E,F) and the Transwell migrated cell count (Figure 4H) confirmed that the migration rate in the 3D-EVs group was not only significantly higher than that of the PBS group but also statistically superior to the 2D-EVs group.

We evaluated the capillary-like tube formation capacity of HUVECs on Matrigel. The PBS-treated HG group showed a disrupted and sparse capillary network. Both 2D-EVs and 3D-EVs treatments promoted the formation of tube-like structures compared to the PBS group (Figure 4I). Quantitative assessment revealed that 3D-EVs significantly upregulated the number of nodes (Figure 4J) and the number of junctions (Figure 4K) compared to both the PBS and 2D-EVs groups, indicating a higher level of vascular network complexity induced by 3D-cultured EVs. While both EV groups increased the total tube length compared to the PBS control, no statistical difference was observed between the 2D-EVs and 3D-EVs groups in this specific parameter (Figure 4L). These findings suggest that while both types of EVs support basic tube elongation, 3D-EVs possess a superior capacity to enhance the branching and structural integration of the nascent vascular network under high-glucose stress.

Taken together, these findings compellingly demonstrate that the 3D FG hydrogel microenvironment not only increases EV yield but also fundamentally enhances their pro-angiogenic potency, making 3D-EVs a superior therapeutic candidate for reversing HG-induced endothelial dysfunction.

### 3.5. 3D-EVs Effectively Modulate Macrophage Polarization from M1 to M2 Phenotype

**Figure 5:**
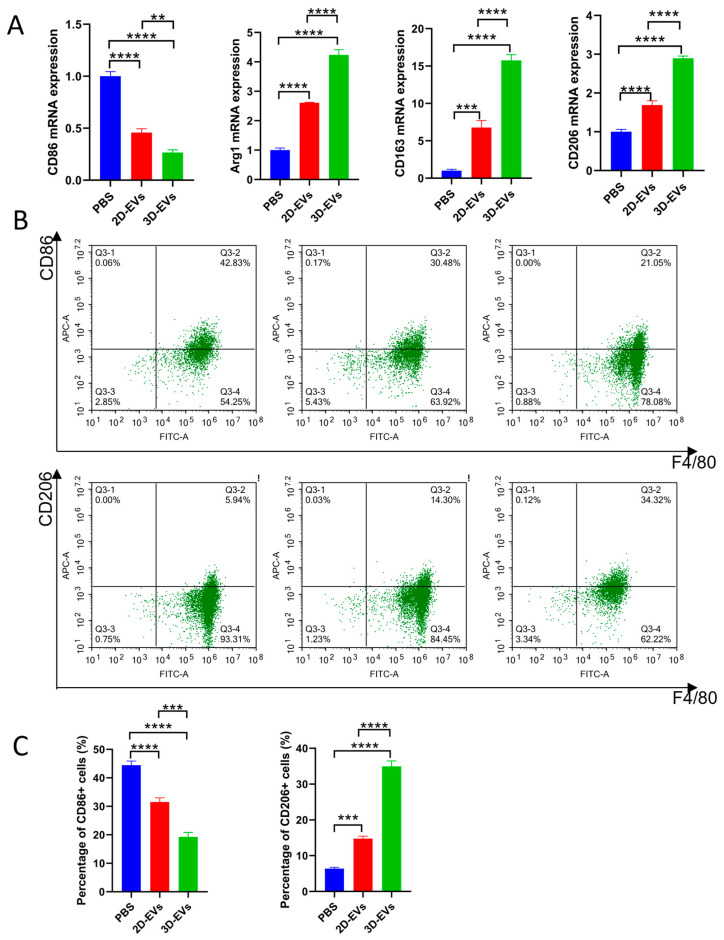
3D-EVs promote the polarization of macrophages from the M1 to M2 phenotype. (**A**) qPCR analysis of M1 marker (CD86) and M2 markers (Arg-1, CD163, and CD206) in RAW264.7 macrophages across different treatment groups (PBS, 2D-EVs, and 3D-EVs). (**B**) Representative flow cytometry plots showing the populations of CD86^+^ (M1) and CD206^+^ (M2) cells. The percentages of positive cells for each group are indicated. (**C**) Quantitative statistical analysis of the percentage of M1 (CD86^+^) and M2 (CD206^+^) macrophages based on flow cytometry data. Data are presented as mean ± SD (*n* = 3, ** *p* < 0.01, *** *p* < 0.001, **** *p* < 0.0001).

To assess the immunomodulatory effects of 2D-EVs and 3D-EVs on macrophages, we examined the expression of polarization markers. qPCR results showed that, compared to the PBS and 2D-EVs groups, 3D-EVs treatment significantly downregulated the expression of the M1 marker CD86, while significantly upregulating the expression of M2-related markers Arg-1, CD163, and CD206 (Figure 5A). Flow cytometry further confirmed this phenotypic shift. The proportion of CD86^+^ (M1) macrophages decreased from 42.83% in the PBS group to 30.48% in the 2D-EVs group, reaching a minimum of 21.05% in the 3D-EVs group, while the proportion of CD206^+^ (M2) macrophages was significantly higher in the 3D-EVs group (34.32%) than in the 2D-EVs group (14.30%) and the PBS group (5.94%) (Figure 5B). Statistical analysis showed that 3D-EVs were significantly superior to 2D-EVs in reducing M1 polarization and promoting M2 polarization (*p* < 0.01) (Figure 5C). These results indicate that three-dimensionally cultured extracellular vesicles have superior immunomodulatory efficacy and can create a favorable regenerative microenvironment for diabetic wound repair by alleviating excessive inflammation.

### 3.6. 3D-EVs Accelerate Wound Healing and Enhance Microvascular Regeneration in Diabetic Mice

**Figure 6:**
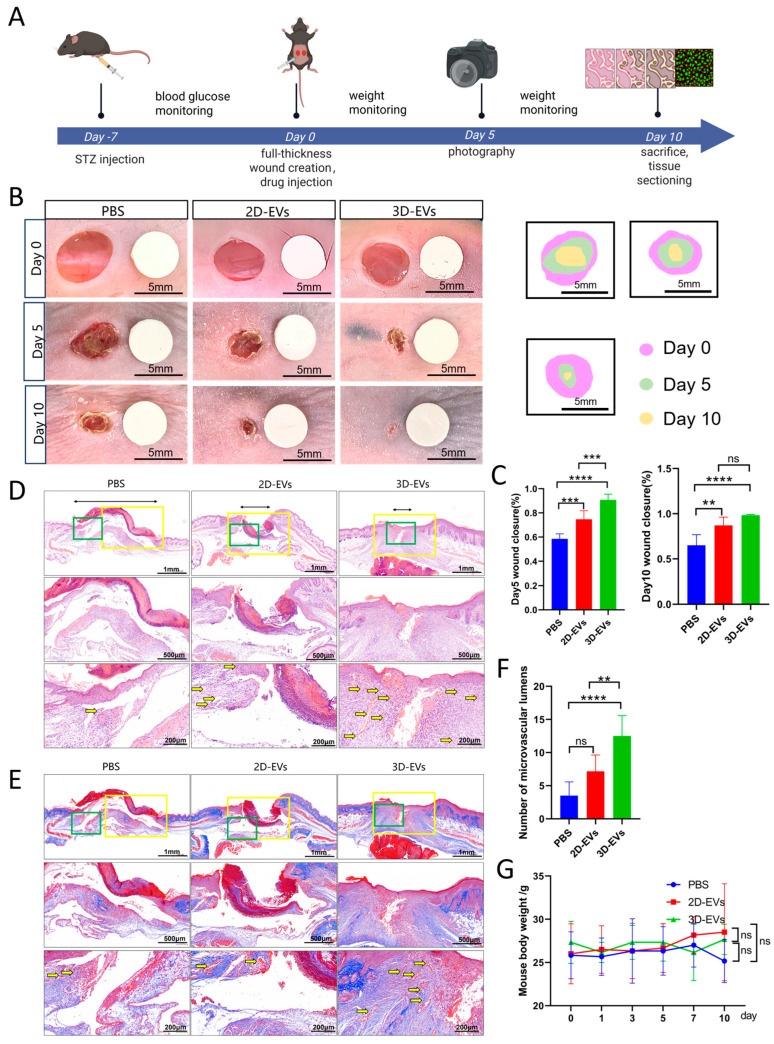
3D-EVs promote wound healing and neovascularization in diabetic mice. (**A**) Schematic diagram of the animal experimental design, treatment groups, and sampling timeline. (**B**) Representative photographs of the skin wounds in the PBS, 2D-EVs, and 3D-EVs groups at days 0, 5, and 10 post-surgery. (**C**) Quantitative analysis of the wound healing rate at day 5 and day 10 (*n* = 6). (**D**,**E**) Histological evaluation of wound sections at day 10 via H&E staining (**D**) and Masson’s trichrome staining (**E**). Top row: Low-magnification panoramic views of the wound area (Scale bar = 1 mm). Middle row (Yellow boxes): Magnified views showing the details of re-epithelialization and epithelial thickness (Scale bar = 500 μm). Bottom row (Green boxes): High-magnification views highlighting microvascular lumina (yellow arrows) and collagen deposition (Scale bar = 200 μm). Yellow arrow: microvascular lumen. (**F**) Quantification of the number of microvascular lumina per high-power field. (**G**) Monitoring of mouse body weight throughout the treatment period. Data are presented as mean ± SD (*n* = 6, ** *p* < 0.01, *** *p* < 0.001, **** *p* < 0.0001, ns shows no significant).

To evaluate the in vivo therapeutic potential of 3D-EVs, we established a full-thickness skin wound model in diabetic mice and treated them with PBS, 2D-EVs, or 3D-EVs, monitoring for 10 days (Figure 6A). Digital imaging of the wound showed that both EV treatments accelerated wound closure (Figure 6B). Quantitative analysis on day 5 showed a stepwise improvement in healing rate: 3D-EVs were superior to 2D-EVs, and both were superior to PBS (*p* < 0.001); by day 10, there was no significant difference between 2D-EVs and 3D-EVs, indicating that both could effectively drive late-stage physical closure (Figure 6C).

Histological analysis on day 10 showed that the 3D-EVs group achieved better re-epithelialization, with more mature and continuous stratified epithelium (Figure 6D,E). Numerous microvascular cavities and dense, well-arranged collagen fibers were observed in the wounds treated with 3D-EVs. Quantitative analysis confirmed that the number of microvessels in the 3D-EVs group was significantly higher than that in the PBS and 2D-EVs groups (*p* < 0.01), while there was no significant difference between the latter two groups (Figure 6F). These results indicate that 3D-EVs not only accelerate physical wound closure but also improve the structural quality of regenerated tissue by promoting mature angiogenesis and collagen remodeling.

No significant difference in mouse body weight was observed among the groups during the experiment, confirming the excellent systemic biocompatibility of EVs treatment (Figure 6G).

### 3.7. 3D-EVs Promote Functional Angiogenesis and Immunomodulation In Vivo

**Figure 7:**
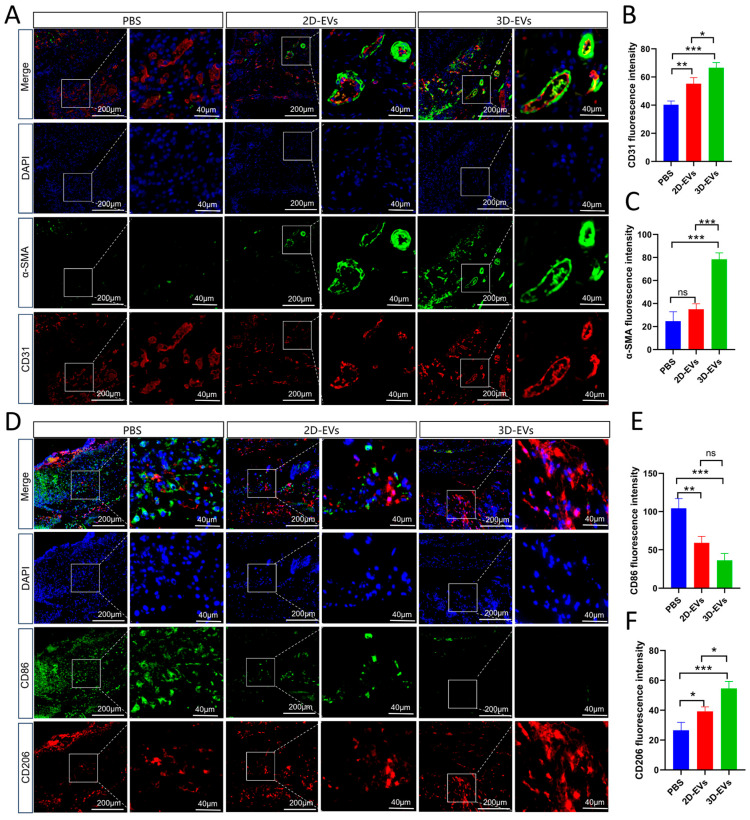
3D-EVs enhance mature angiogenesis and modulate macrophage polarization in diabetic wounds. (**A**) Representative immunofluorescence images of wound sections stained for CD31 (red), α-SMA (green), and DAPI (blue). (**B**,**C**) Quantitative analysis of the fluorescence intensity for CD31 (**B**) and α-SMA (**C**). (**D**) Representative immunofluorescence images of wound sections stained for CD206 (red), CD86 (green), and DAPI (blue). (**E**,**F**) Quantitative analysis of the fluorescence intensity for CD86 (**E**) and CD206 (**F**). Data are presented as mean ± SD (*n* = 3, * *p* < 0.05, ** *p* < 0.01, *** *p* < 0.001, ns shows no significant).

To further elucidate the mechanisms by which 3D-EVs accelerate diabetic wound healing, we performed dual-immunofluorescence staining on wound sections at day 10. We first evaluated the neovascularization within the granulation tissue by co-staining for CD31 (a marker for vascular endothelial cells) and α-SMA (a marker for smooth muscle cells/pericytes) (Figure 7A). The 3D-EVs group exhibited the highest density of CD31+ microvessels. Quantitative analysis of CD31 fluorescence intensity (Figure 7B) confirmed significant differences among all three groups (*p* < 0.05), with 3D-EVs demonstrating the most potent pro-angiogenic effect. Notably, the α-SMA intensity (Figure 7C) was significantly higher in the 3D-EVs group compared to both the PBS and 2D-EVs groups, whereas no statistical difference was found between the PBS and 2D-EVs groups. The high co-localization of CD31 and α-SMA in the 3D-EVs group indicates not only an increase in vessel number but also enhanced vascular maturation and stabilization.

Furthermore, we examined the macrophage polarization status in situ by co-staining for CD86 (M1 marker) and CD206 (M2 marker) (Figure 7D). The results showed that EV treatments effectively shifted the macrophage population from a pro-inflammatory to a pro-regenerative phenotype. Quantitative analysis of CD86 intensity (Figure 7E) showed that both 2D-EVs and 3D-EVs significantly reduced M1 infiltration compared to the PBS group, although no significant difference was observed between the two EV groups. Crucially, the CD206 fluorescence intensity (Figure 7F) was markedly and significantly higher in the 3D-EVs group compared to all other groups (*p* < 0.05). These findings demonstrate that while both EV types can suppress inflammation, 3D-EVs possess a superior capacity to actively induce M2 macrophage polarization in the diabetic wound microenvironment, thereby creating a favorable milieu for tissue repair.

## 4. Discussion

**Figure 8:**
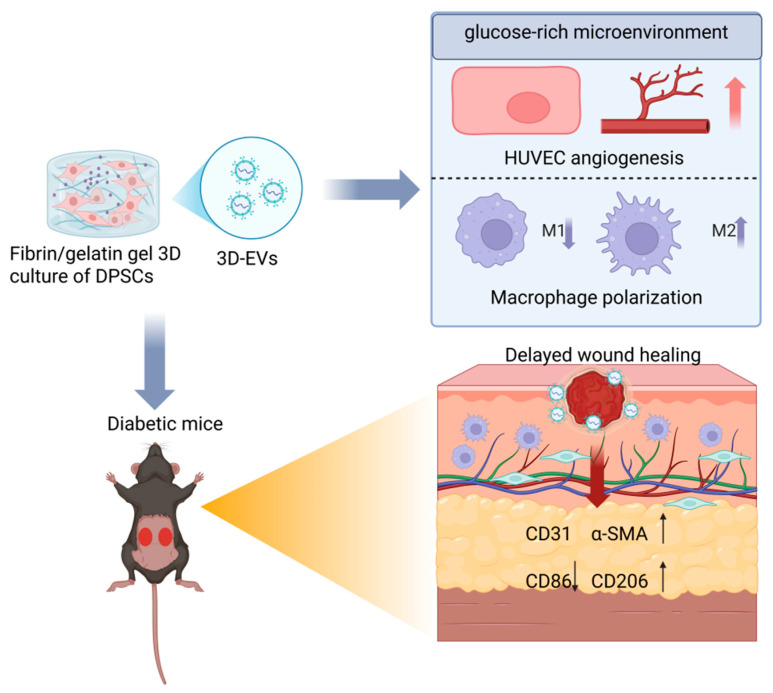
Schematic illustration of the 3D fibrin/gelatin (FG) hydrogel system for enhancing the therapeutic potency of DPSC-EVs in diabetic wound healing.

In this study, we successfully developed a 3D biomimetic fibrin/gelatin hydrogel system. This system not only significantly increased the production yield of DPSC-EVs but also fundamentally improved their biological functions compared to traditional 2D cultures. Our experimental results demonstrate that 3D extracellular vesicles (3D-EVs) possess a superior capacity to correct endothelial dysfunction induced by hyperglycemia and to promote the polarization of macrophages from the pro-inflammatory M1 phenotype to the pro-regenerative M2 phenotype. These synergistic effects effectively accelerated wound healing and improved the quality of tissue regeneration in diabetic mice. Therefore, 3D-EVs represent a highly effective and promising cell-free therapy for the management of chronic diabetic wounds (Figure 8).

A major challenge in the clinical use of extracellular vesicle (EV) therapy lies in the low yield and inconsistent bioactivity of traditional two-dimensional (2D) culture systems. Our study shows that using a fibrin/gelatin (FG) hybrid hydrogel as a three-dimensional (3D) scaffold effectively addresses these issues. It significantly increases both the quantity and therapeutic quality of EVs derived from DPSCs. The substantial increase in EV production in the 3D system is likely due to the hydrogel’s biomimetic structure. Unlike the flat, rigid surfaces of 2D culture flasks, the microporous network of the FG hydrogel offers a much higher surface area to volume ratio. This allows for higher cell density and improved cell–matrix interactions. The hydrogel’s mechanical environment is crucial for EV biogenesis. The 3D matrix provides spatial confinement and rigidity that transmits physical signals to cells. These mechanical signals are transmitted by the cytoskeleton to endosomes. This process can accelerate the maturation of multivesicular bodies (MVBs) and their fusion with the cell membrane, ultimately increasing the release of vesicles into the extracellular space [34,35].

In addition to the increased number, the biological functions of these extracellular vesicles were fundamentally altered by the three-dimensional environment. Under high glucose stress, 3D-EVs showed a significantly stronger ability to restore cell function compared to 2D-EVs. We also observed that while both 2D-EVs and 3D-EVs promoted endothelial cell elongation and migration, 3D-EVs significantly increased the number of vascular branches and junctions, a difference that is biologically significant. In tissue engineering, the density of vascular branch points is often a more reliable indicator of mature, connected capillary networks than vascular length [36]. This functional improvement suggests that three-dimensional culture stimulates the secretory activity of dental pulp stem cells. Numerous studies have shown that three-dimensional pretreatment in biomimetic matrices can modify extracellular vesicle content, thereby inducing the production of microRNAs and regenerative proteins that are more specific to tissue repair [37,38,39]. Consistent with these findings, preliminary high-throughput sequencing conducted by our team also revealed that extracellular vesicles derived from dental pulp stem cells cultured in three dimensions exhibited different microRNA expression signatures compared to 2D-EVs [40]. Three-dimensional culture induced strong overexpression of several key microRNAs, such as miR-126-3p, miR-210, and miR-146a-5p, which are recognized mediators of endothelial cell survival, pro-angiogenic signaling, and inflammation relief.

The significant increase in the number of CD31-labeled periendothelial α-SMA-positive cells in the 3D-EVs group indicates increased recruitment of pericytes or smooth muscle cells, a process known as vascular wrapping, which is crucial for preventing the degeneration of newly formed fragile vessels, a common cause of treatment failure in diabetic wounds [41,42]. Unlike conventional 2D-EVs, which showed limited pericyte recruitment in our experimental model, 3D-EVs appear to activate specific signaling pathways that are essential for coordinating complex interactions between different types of vascular cells, thereby promoting the structural maturation and stabilization of newly formed microvessels [43]. These results suggest that the three-dimensional fibrin/gelatin environment strategically increases the concentration of specific bioactive molecules in the contents of extracellular vesicles, which are crucial for overcoming the pathological barriers to healing diabetic wounds.

The superior immunomodulatory capacity of 3D-EVs compared to 2D-EVs is a key finding of this research. In a hyperglycemic environment, both types of EVs reduced M1 markers, but 3D-EVs proved much more effective at increasing specific M2 markers such as CD206 and Arg-1. This difference is crucial because M2 macrophages are not only anti-inflammatory, but they are also active orchestrators of tissue repair, releasing important growth factors such as VEGF and TGF-β [44,45,46]. The strong polarization toward the M2 phenotype observed in situ suggests that 3D-EVs can effectively transform the diabetic wound bed into a pro-regenerative environment. This enhanced immunomodulatory mechanism is likely due to the unique molecular content of 3D-EVs during the hydrogel priming process. Our group’s previous high-throughput sequencing revealed that 3D culture significantly enriches DPSC-EVs with miR-146a-5p, a potent anti-inflammatory microRNA that specifically targets the TRAF6/IRAK1 signaling axis. This selective enrichment suggests that 3D-EVs modulate macrophage polarization by suppressing the NF-κB signaling pathway, thereby facilitating the transition from a pro-inflammatory M1 phenotype to a pro-healing M2 phenotype in the diabetic wound microenvironment [39]. Furthermore, the synergy between inflammation resolution and neovascularization, frequently termed immuno-angiogenesis, is particularly evident in our model. By promoting M2 polarization, 3D-EVs not only reduce tissue damage from excessive inflammation but also indirectly support the maturation of the microvasculature, thereby creating a self-sustaining cycle of repair [47].

Despite these encouraging results, several limitations warrant further investigation. While our miRNA sequencing revealed differential enrichment of specific miRNAs in 3D-EVs, a comprehensive comparison of protein, lipid, and other RNA species between 2D-EVs and 3D-EVs was not performed and represents an important direction for future mechanistic studies. Additionally, the long-term safety and stability of DPSC-EVs in larger animal models should be evaluated before proceeding to clinical trials. Future research focusing on these mechanistic insights and long-term efficacy will be crucial for the successful translation of this 3D-EV delivery system into standard clinical practice for diabetic regenerative medicine. We acknowledge that ultracentrifugation does not fully separate exosomes from other sEV subtypes. Therefore, the functional differences observed between 2D-EVs and 3D-EVs reflect the total EV population from each culture condition. Future studies employing density gradient purification or single-vesicle analysis are needed to determine whether specific EV subtypes contribute differentially to the therapeutic effects. The in vitro degradation assay was performed in PBS, which does not fully replicate the enzymatic environment present in blood or wound fluid. Therefore, future studies should employ plasma or MMP-containing buffers to obtain more physiologically relevant degradation profiles.

We also acknowledge that splints were not used in our mouse wound model; therefore, wound closure occurred primarily by contraction, which differs from human wound healing, where re-epithelialization and granulation tissue formation are more critical. Nonetheless, our histological assessments of re-epithelialization, angiogenesis, and immune modulation provide evidence of regenerative effects beyond contraction. Future studies using splinted wound models are needed to better recapitulate human diabetic wound healing.

## 5. Conclusions

In summary, we successfully established a biomimetic 3D fibrin/gelatin hydrogel system that significantly enhances the yield and therapeutic potency of DPSC-derived EVs. Compared to conventional 2D-EVs, 3D-EVs exhibit superior capacity in promoting functional, mature angiogenesis and driving the polarization of macrophages toward a pro-regenerative M2 phenotype. These synergistic effects effectively resolve the persistent inflammation and impaired vascularization characteristic of diabetic microenvironments, leading to accelerated and high-quality wound healing in vivo. Our findings highlight the 3D FG hydrogel as an efficient platform for EV production and position 3D-EVs as a promising cell-free therapeutic strategy for the clinical management of chronic diabetic wounds.

## Figures and Tables

**Table 1:** List of specific primers.

Gene	Sequence (5′-3′)
*GAPDH*	F: AGGTCGGTGTGAACGGATTTG R: GGGGTCGTTGATGGCAACA
*CD86*	F: TGTGTCTGTTCAAACGCTTCATC R: CCCTTGCCAGTGATGAGGAG
*Arg1*	F: CTCCAAGCCAAAGTCCTTAGAG R: AGGAGCTGTCATTAGGGACATC
*CD163*	F: TCCACACGTCCAGAACAGTC R: CCTTGGATTCTGGTGGGCAG
*CD206*	F: TCTTTTGCCTTTGGCTGGGG R: AATGTTCCTTTTGTCCGCCG

## Data Availability

The original contributions presented in the study are included in the article. Further inquiries can be directed to the corresponding author.
